# Combination of cyclophosphamide and double-stranded DNA demonstrates synergistic toxicity against established xenografts

**DOI:** 10.1186/s12935-015-0180-6

**Published:** 2015-03-19

**Authors:** Ekaterina A Alyamkina, Valeriy P Nikolin, Nelly A Popova, Alexandra M Minkevich, Artem V Kozel, Evgenia V Dolgova, Yaroslav R Efremov, Sergey I Bayborodin, Oleg M Andrushkevich, Oleg S Taranov, Vladimir V Omigov, Vladimir A Rogachev, Anastasia S Proskurina, Evgeniy I Vereschagin, Elena V Kiseleva, Maria V Zhukova, Alexandr A Ostanin, Elena R Chernykh, Sergey S Bogachev, Mikhail A Shurdov

**Affiliations:** Institute of Cytology and Genetics, Siberian Branch of the Russian Academy of Sciences, 10 Lavrentieva ave, 630090 Novosibirsk, Russia; Novosibirsk State University, Novosibirsk, 630090 Russia; The State Research Center of Virology and Biotechnology VECTOR, Koltsovo, Novosibirsk region 630559 Russia; Novosibirsk State Medical Academy, Novosibirsk, 630091 Russia; Institute of Clinical Immunology, Siberian Branch of the Russian Academy of Medical Sciences, Novosibirsk, 630099 Russia; LLC Panagen, Gorno-Altaisk, Russia

**Keywords:** Ascites Krebs-2, Cyclophosphamide, Double-stranded DNA, Apoptosis, Necrosis, Sepsis, MCF-7

## Abstract

**Background:**

Extracellular double-stranded DNA participates in various processes in an organism. Here we report the suppressive effects of fragmented human double-stranded DNA along or in combination with cyclophosphamide on solid and ascites grafts of mouse Krebs-2 tumor cells and DNA preparation on human breast adenocarcinoma cell line MCF-7.

**Methods:**

Apoptosis and necrosis were assayed by electrophoretic analysis (DNA nucleosomal fragmentation) and by measurements of LDH levels in ascitic fluid, respectively. DNA internalization into MCF-7 was analyzed by flow cytometry and fluorescence microscopy.

**Results:**

Direct cytotoxic activity of double-stranded DNA (along or in combination with cyclophosphamide) on a solid transplant was demonstrated. This resulted in delayed solid tumor proliferation and partial tumor lysis due to necrosis of the tumor and adjacent tissues. In the case of ascites form of tumor, extensive apoptosis and secondary necrosis were observed. Similarly, MCF-7 cells showed induction of massive apoptosis (up to 45%) as a result of treatments with double-stranded DNA preparation.

**Conclusions:**

Double-stranded DNA (along or in combination with cyclophosphamide) induces massive apoptosis of Krebs-2 ascite cells and MCF-7 cell line (DNA only). In treated mice it reduces the integrity of gut wall cells and contributes to the development of systemic inflammatory reaction.

**Electronic supplementary material:**

The online version of this article (doi:10.1186/s12935-015-0180-6) contains supplementary material, which is available to authorized users.

## Background

Double-stranded extracellular DNA fragments of either exogenous or endogenous origin act as key regulators and active participants of various processes in an organism. First, exogenous nucleic acids serve as pathogen-associated molecular patterns and activate different cells of immune system geared towards pathogen elimination. Endogenous extracellular double-stranded DNA (dsDNA) may also function as an activator of adaptive immune response [1-8]. Additionally, endogenous extracellular dsDNA present in the blood plasma and interstitial fluids reflects the functional state of an organism and may serve as a marker of ongoing disease [9-12]. It is well-established that dsDNA is an inducer of autoimmunity and that it may also mediate the bystander effect [13-19]. Systemic inflammatory reaction and sepsis are normally accompanied with rapid increase in serum DNA concentration [18,20-24]. On a cellular level, dsDNA fragments reaching the cell internal comparments may participate in a number of processes. First, the very presence of extracellular DNA fragments in the cell results in cell cycle arrest and induces DNA repair [25-27]. Under certain conditions, dsDNA fragments may actively interfere with accurate progression of DNA repair [28-30]. DsDNA fragments delivered to the cytoplasm of somatic cells are detected by a variety of cytosolic sensors thereby launching the first steps of adaptive immune response [31-35]. Data are also available demonstrating that exogenous circulating DNA may participate in transport of genetic material from destroyed cancer cells to normal cells, thereby mediating their malignant transformation. A special term, “genometastasis”, was coined to describe this phenomenon [36,37]. Nucleic acids, including dsDNA, have been shown to be present in exosomes. Such nucleic acids were hypothesized to serve as an “internal standard” of the organism functional state, which helps certain cell populations tune in their physiological and molecular processes. Presently, mounting evidence suggests that extracellular nucleic acids, including dsDNA, in fact represent a novel regulatory module controlling a plethora of cellular processes [38-48].

Our studies establish for the first time the direct cytotoxic activity of dsDNA preparation administered alone or in combination (and in synergy) with a cytostatic drug cyclophosphamide (CP) against cancer cells of various origin. This treatment results in massive apoptosis of tumor cells, which is however uncoupled from internalization of dsDNA fragments into the cell interior.

## Results

### Motivation of the study

Our earlier studies of how extracellular exogenous dsDNA contributes to the activation of professional properties of antigen-presenting dendritic cells and establishment of anticancer CD8 + −mediated antitumor immunity reported a number of puzzling effects unrelated to the development of immune response. Namely, we observed significantly suppressed proliferation of tumors grafts in either solid or ascites forms [49-54]. We found that upon consecutive treatment with CP and dsDNA, there is a short period of time after CP injection when administration of dsDNA causes direct toxicity to the cells of engrafted tumor. Three formally plausible explanations could account for such an effect resulting in delayed growth of engrafted tumors. First, this could occur due to selective DNA internalization by cancer cells, followed by interference with repair of CP-induced interstrand cross-links (ICLs), essentially in the same way as it was observed for mouse hematopoietic stem cells [28,29]. Second, this could be caused by a yet unknown form of interaction between DNA and an eukaryotic cell which occurs without dsDNA internalization (analogous to the bystander effect) [19,55]. Finally, the effector of direct tumor toxicity could be not the dsDNA preparation itself, but rather cross-linked dsDNA. One of the treatment regimens was consistent with such a possibility, as it involved simultaneous presence of extracellular dsDNA fragments and a CP metabolite, phosphoramide mustard, in the organism. The latter compound is an alkylating metabolite of CP, which is formed upon CP hydrolysis in the liver, and so it may induce formation of ICLs in dsRNA and dsDNA molecules. Given that phosphoramide mustard easily gets transported to all the cells of an organism, one can expect that injected dsDNA will be inevitably cross-linked.

### Pilot experiments that defined the study design

**Figure 1:**
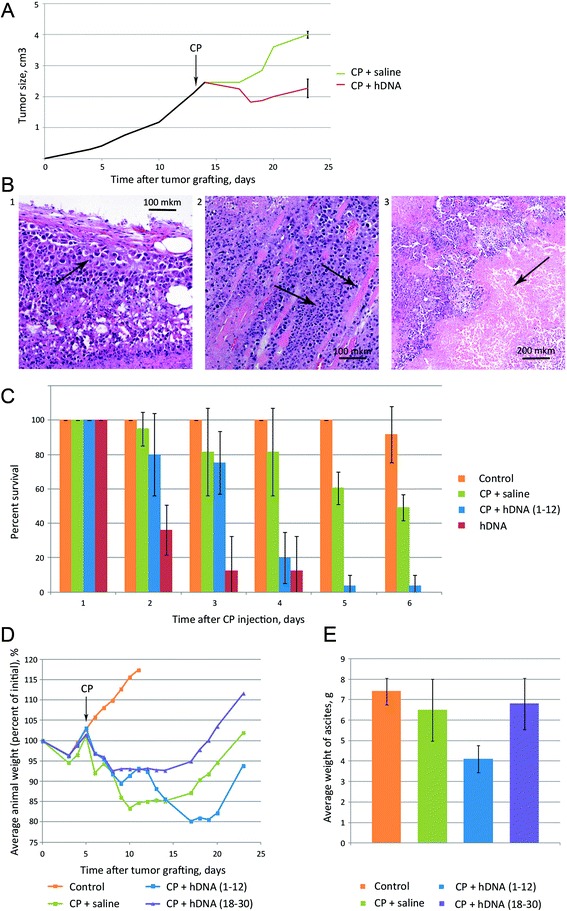
**Effects of CP and/or hDNA treatments on Krebs-2 tumor cells propagated as ascites or solid tumors. А)** Growth of solid Krebs-2 tumors in animals treated with CP followed with hDNA or saline injections (n = 4). Time of CP injection is denoted by an arrow. **B)** Representative images of tumor sections after the treatments: 1 – tumor tissue is clearly distinct from the dermal elements and subcutaneous tissue, as it displays pronounced cellular atypia and aggressive growth (arrow); 2 – tumor cell fibers interleave with fragments of muscle fibers (arrows); 3 – massive necrosis of tumor tissue (arrow). **С)** Percentage of surviving animals bearing Krebs-2 ascites and treated with hDNA, CP + saline or CP + hDNA 1–12 hours post CP injection (number of experiments, n = 3-5). Survival in the groups was compared daily. **D)** Changes in weight of animals engrafted with Krebs-2 ascites after CP + saline or CP + hDNA 1–12 or 18–30 hours post CP injection. Initial weight of animals was taken as 100%. Average weight of mice is shown (n = 8). **Е)** Comparison of ascites weight in animals treated with CP + saline vs CP + hDNA (1–12 or 18–30 hours post CP treatment), on day 8 after the treatments (n = 8).

To better understand the mechanism of how CP + dsDNA treatment suppresses tumor graft growth, we proceeded to perform a series of pilot experiments. First, using a solid form of Krebs-2 tumor, we directly evaluated the synergistic toxicity of CP and human dsDNA (hDNA). DNA was injected directly into tumors at 1–12 h and 18–30 h timepoints post-CP (as was described in [28]), so as to match the timing of ICL repair phases, NER and homologous recombination. Experiments were performed multiple times and results from a typical experiment including pathology analysis of mouse organs and tissues are presented below. During the 9 days after the therapy, the tumors would stop growing and remained ~2.0 cm^3^ (CP + hDNA), or progressed from ~2.0 to 4.0 cm^3^ upon CP + saline treatment (Figure 1А). Mice treated with just hDNA consistently displayed lysis of tumor graft and adjacent tissues, which was accompanied by profuse purulent discharge. It must be emphasized that significant mortality was observed in the CP + hDNA groups of mice bearing solid Krebs-2 transplants during the days 1–4 following the treatment (data not shown). These observations are consistent with massive histolysis at injection sites, first and foremost with decay of tumor tissue accompanied with intoxication that is ultimately lethal.

To approach the possible causes of these destructive effects and to understand why experimental animals would succumb almost synchronously, we first performed pathology and morphology analysis of organs and tumor foci from animals treated with CP + saline or CP + hDNA. Animals with a tumor of ~2.5 cm^3^ received CP injection (200 mg/kg), which was followed with 12 injections of hDNA (250 mkg each) or saline every hour, straight into the tumor graft. On day 3 after these treatments, one animal was sacrificed from both experimental and control groups, two more and one more were sacrificed on days 4 and 5. All animals from the experimental group were in moribund state.

### Pathology report of mouse necropsies

All experimental animals displayed pronounced pathological changes in the liver and spleen. Focal degeneration of hepatocytes of a granular or ballooning type was observed in the liver. Inflammatory reaction generally manifested as increased numbers of lymphocytes in blood capillaries. In several cases, we observed formation of predominantly round cell infiltrations around portal area and central veins. No evidence of liver parenchyma necrosis was found in any of the animals. All mice displayed marked reduction in size of spleen lymphoid follicles.

Notably, moderate dystrophy of myocardium was evident in a single tumor-bearing mouse treated with CP + hDNA and one more mouse from the same group had a subepicardial metastasis-like lesion. The rest of the experimental animals displayed no pathological changes in myocardium or other organs.

Tumor tissue was clearly distinct from the surrounding dermal elements and subcutaneous tissue due to its pronounced cellular atypia and aggressive growth pattern. The latter phenotype was particularly notable where fibers of tumor cells interleaved with fragments of muscle fibers. Massive necrosis of tumor tissue was particularly strong in the group of CP + hDNA-treated animals consistent with their symptoms. Polymorphocellular infiltrate formed a thin border between the tumor and healthy tissues (Figure 1B, Additional file 1). Pathology-wise, the highest organ toxicity was observed in animals treated with a combination of CP and multiple ICL-hDNA injections (Additional file 1).

To avoid the lethality concern and to determine the most efficacious schedule of CP + hDNA administration, we performed a series of experiments wherein mice with tumor grafts of just ~0.25 cm^3^ were treated. This modification to the treatment protocol resulted in that lysed tumor material was effectively cleared by immune cells, thereby avoiding lethal toxicity. The highest degree of cytoreductive activity was observed when mice were treated with hDNA during the period of 1–12 h post-CP injection (data not shown).

Solid Krebs-2 tumors are densely cellularized; they are typically enclosed in a connective tissue capsule and lack extensive capillary network. Clearly then, delivery of any high-molecular weight molecules (such as human dsDNA) to such tumors is very inefficient and non-uniform [49] This obstacle prevented us from directly assessing the tumor response to CP + hDNA therapy and prompted the development of an alternative model, where the same Krebs-2 tumor cell line is used, yet it is engrafted to mice as an ascites form.

We first attempted to quantify regression of ascites in mice treated with CP or CP + hDNA on days 8–10 after engraftment. Similarly to what was observed with a solid tumor model, such animals succumbed rapidly and synchronously. Hence, no statistically significant analysis of tumor graft size dynamics could be done. Representative survival data are shown in Figure 1C. Next, we established that mice bearing 4–5 day ascites and treated with CP or hDNA, overall do much better. To illustrate this, we present the data from one of the experiments which involved male CC57BR mice who received intraperitonially (i.p.) injection of ascites form of Krebs-2 tumor and then intravenously injected with 200 mg/kg CP on day 6. Of these mice, one subgroup subsequently received 500 mkg/injections of hDNA (i.p. for 12 hours). The other subgroup was similarly administered the same hDNA injections, however those were done 18 hours after CP. Mice from the third subgroup were injected with saline every two hours throughout 0–30 hrs post CP injection. Mouse weight dynamics expressed in relative percentage values is presented in the Figure 1D. Treatment with hDNA preparation during the first twelve hours after CP injection (first subgroup) resulted in a significant delay in proliferation of ascites tumor grafts. Injections during the period of 18–30 hours post CP treatment enhanced ascites growth, as compared to CP + saline and CP + hDNA (1–12) groups (detailed explanation of this effect is presented in [30]).

The effects of treatments were independently assessed in another arm of this experimental series by measuring the ascites weight in animals 8 days after the start of experiment (Figure 1E). We show that treatment with hDNA 1–12 hours post CP injection results in a statistically significant reduction of ascites weight in treated animals.

These pilot experiments involving ascites and solid forms of Krebs-2 tumors indicated four pronounced trends:

1. Very rapid and largely synchronous death of experimental animals was observed, when mice with large tumor burden were treated with a combination of CP and hDNA (regardless of whether the tumor graft was solid or ascitic).
2. Ascites weight decreased and solid tumor tissue lysed in animals treated with CP and hDNA.
3. The strongest therapeutic effect was found when animals received hDNA injections during the first 12 hours post CP injection.
4. Under such regimen (CP + hDNA (1–12)), prolonged regression of ascites growth and delayed proliferation of solid grafts were observed.

Thus, the experimental design of this study aimed at developing the strategy that would allow discriminating the contributions of each of the effects observed and to pinpoint the possible molecular and physiological mechanisms involved. Two major research directions were considered. The first direction focused on understanding why experimental animals succumbed so rapidly and synchronously and why ascites shrank and solid tumors displayed delayed growth. The second direction of research addressed the question of why prolonged remission was observed in ascites-bearing animals treated with CP and hDNA (1–12). Specifically, we analyzed the effects on Krebs-2 tumor-initiating stem cells, which we recently described as capable of naturally internalizing extracellular dsDNA fragments. Most of the experimental data supporting the role of tumor-initiating stem cells elimination in the loss of tumorigenic properties of tumor grafts (and hence in remission of the experimental animals) are presented in [30].

### Comprehensive analysis of pathological processes that develop in synchronously dying experimental animals

The events observed in our pilot experiments defined the routes of follow-up studies. All the subsequent experiments used only ascites form of Krebs-2 tumor. We calculated mouse survival, quantified viable ascitic cells by FACS, and determined the extent of apoptosis (by measuring chromatin fragmentation into nucleosomal ladders). We assessed the degree of necrosis (LDH levels in ascitic fluid), performed pathology and anatomy analysis of tissues, organs, tumor foci and peritoneal wall from ascites-engrafted mice. Additionally, we described the interplay between the ascites size, treatment regimen and sterility of ascitic fluid. Multiple replicas were performed for all of these experiments, and the results of such experiments are presented in the figures. We report a previously undescribed phenomenon of massive apoptosis of Krebs-2 ascites induced by treatment with hDNA without CP preconditioning. Sampling timepoints differed between the experiments depending on the specific goals. The effects of hDNA and ICL-hDNA were also compared.

Experiments performed can be broadly categorized into two groups. The first set of experiments characterized the events occurring in mice upon synergistic action of CP and hDNA preparations. The second set of experiments focused on events in mice treated solely with hDNA.

Results of the first set of experiments are summarized below:

1. When using mice with 9–15 day ascites, the animals in the groups CP + hDNA (1–12), CP + hDNA (18–30), CP + ICL-hDNA (1–12) and CP + ICL-hDNA (18–30) died on days 1–3 after CP injection. With only 1–2 surviving animals, no statistical analysis was possible, and no additional data could be retrieved (Figure 1C).
2. Shrinking of solid or ascites tumors is correlated with massive, up to 80%, apoptosis of cancer cells induced by the treatments (Figure 2A,В). Cancer cell necrosis is also noticeable. Elevated LDH levels (3-6-fold) are observed across the sampling timepoints relatively to the initial values (Figure 2C). The timing of massive apoptosis and surging LDH levels immediately precede the death of experimental animals.

**Figure 2:**
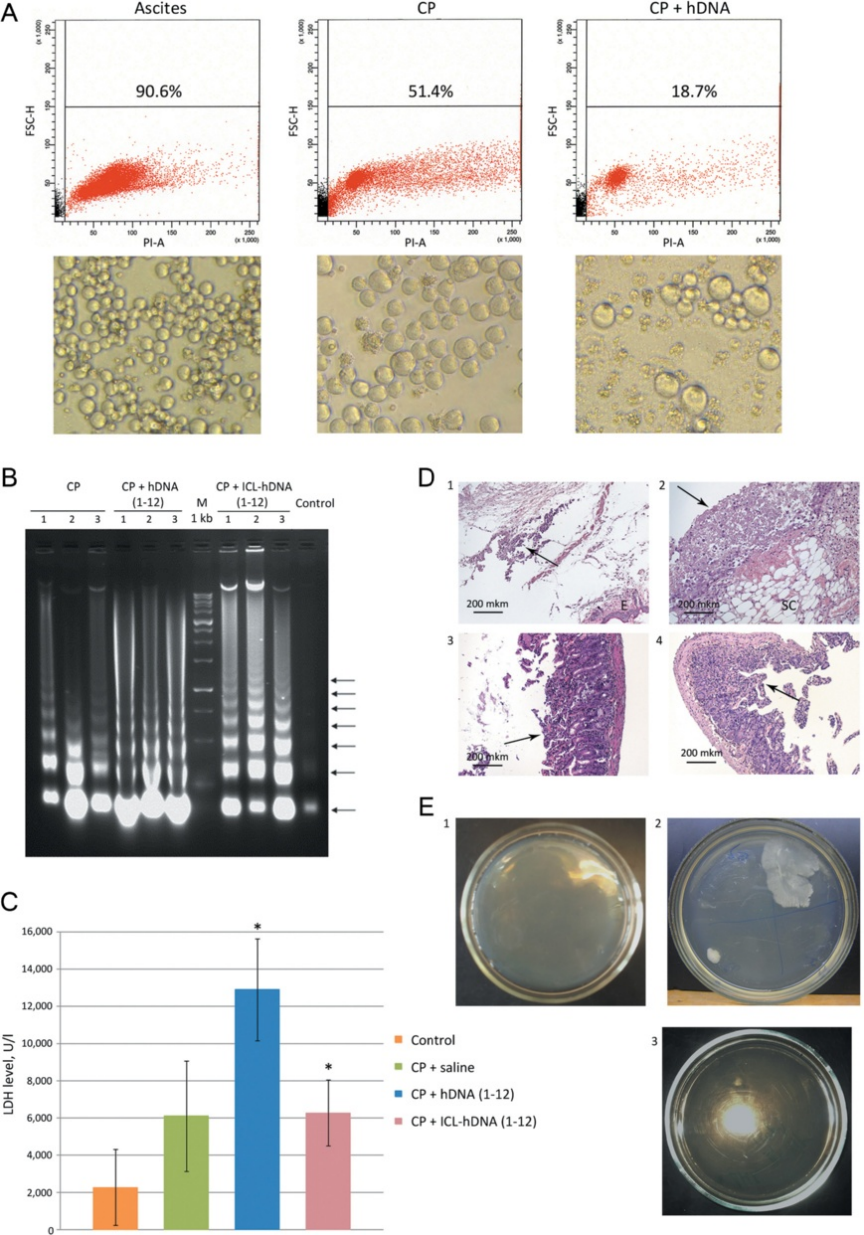
**Apoptosis, necrosis and destructive changes in gut cell wall of tumor-engrafted animals under different treatment regimens. A)** Viability of Krebs-2 ascites isolated from animals treated with CP and/or hDNA. Flow cytometry plots are presented, and percent of viable cells is indicated (top panel). Microscopy analysis of Krebs-2 ascites following different treatments (bottom panel). **B)** Progression of apoptosis assayed as the degree of fragmentation of nucleosomal DNA isolated from ascitic fluid (ascites cells removed) on day 3 after CP + hDNA or CP + ICL-hDNA treatment. Typical apoptotic DNA ladder of nucleosome-size fragments (arrows) is present in DNA from ascitic fluid of treated, but not untreated animals (control). Numbers 1–3 denote ascitic DNA samples obtained from three individual animals. **C)** Degree of necrosis as assayed by LDH levels in ascitic fluid on day 3 after the treatments indicated (P < 0.05, Mann–Whitney U-test, n = 3-6). **D)** Pathology analysis of several affected tissues from animals treated with hDNA preparations and CP: 1 – abdominal wall skin: subdermal necrosis focus (arrow), E – epidermis; 2 – magnified view of necrosis focus: bottom right – subcutaneous tissue (SC), top left – necrotic detritus (arrow); 3 – intestinal mucosa villi appear rounded as a result of ongoing destructive process (arrow), epithelium is almost entirely gone, stroma appears swelled, with infiltrating lymphocytes present. Taken together, these features are consistent with a pathomorphology pattern of acute enteritis; 4 – extensive mucosal swelling, remnants of villi and pronounced inflammatory infiltration with lymphoid cells. Arrow points to sclerotic mucosa (section of normal intestine is shown in Additional file 1). These features are morphologically compatible with the signs of chronic enteritis. **E)** Sterility cultures of hDNA preparation and ascites grown in mice: 1 – 5-day ascites; 2 – 9-day ascites; 3 – hDNA (1 mg total).
3. Pathology analysis suggests that when ascites-bearing mice are treated with a combination of CP and hDNA, necrosis is induced in both the target tumor and the surrounding non-tumor tissues, particularly in peritoneum. Large-scale destructive changes are present in the intestinal wall, which may compromise its integrity thereby affecting its permeability (Figure 2D). Increased gut wall permeability likely results in rapid infestation of ascitic fluid by intestinal bacteria.
4. By day 5, ascites fluid remains sterile. We show that by days 9–15, 75% of ascites samples are non-sterile and are infected with bacterial flora. This bacterial infestation is not due to bacterial contamination of DNA preparation, as no bacteria are detectable in 10 mg (100 mkl) of hDNA prep (Figure 2E). The total amount of hDNA received by each mouse in course of experiments is 6 mg.
5. Combined CP + hDNA treatment leads to infection of ascitic fluid within 24 hours, and is correlated with the death of experimental animals. In contrast, a series of saline injections following CP administration does not result in instantaneous infection of ascitic fluid and rapid death of mice.

**Figure 3:**
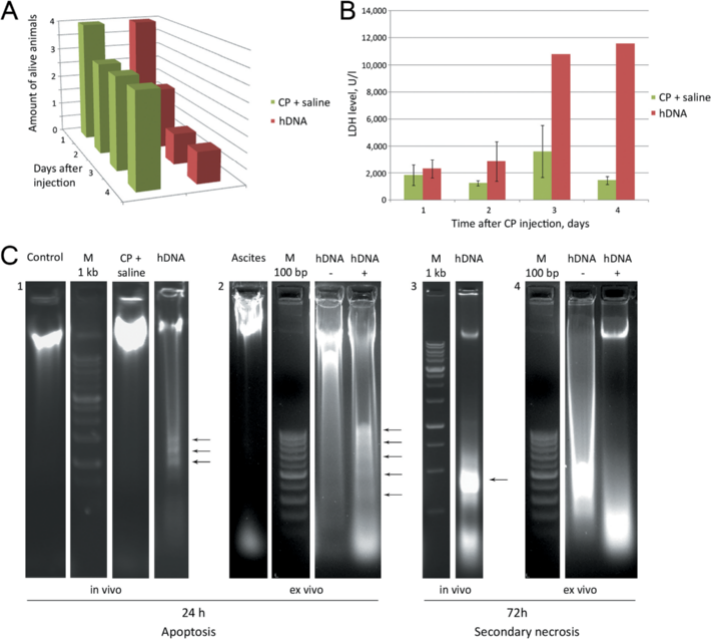
**Analysis of apoptosis induction in ascitic cells 24 or 72 hours after first hDNA injections. А)** Survival of Krebs-2 ascites-engrafted animals after hDNA or CP + saline treatments. **B)** LDH levels measured in ascitic fluid collected from hDNA or CP + saline groups of mice (n = 4-5). **C)** Electrophoretic separation of DNA isolated from ascitic fluid of mice 24 and 72 hours after injections of hDNA (in vivo) or from culture medium following 24 or 72 hours incubation of ascites with hDNA (ex vivo) 1 – Control lane shows DNA of untreated Krebs-2 ascites, hDNA lane represents ascitic fluid DNA isolated 24 hours post direct hDNA injections into ascites; 2 – Ascites – DNA from intact ascites, hDNA- and hDNA + − 24 hour incubation of Krebs-2 ascites in the absence (−) or presence (+) of hDNA, respectively. Arrows on the hDNA+ lane show characteristic pattern of nucleosomal fragmentation of chromatin; 3 – hDNA – DNA isolated from ascitic fluid 72 hours after hDNA injections directly into ascites grafts. Arrow indicates specific DNA band originating from necrotic cells; 4 – hDNA- and hDNA + − incubation of Krebs-2 ascites for 72 hours in the absence or presence of hDNA preparation, respectively. By 24 hours, apoptotic pattern is already noticeable, whereas by 72 hours it transitions into the pattern characteristic of secondary necrosis.
- Our data suggest the CP blocks induction of apoptosis by hDNA preparation, which appears as absence of nucleosomal ladder that is typical for DNA monotherapy (data not shown). In contrast, in mice treated with just CP, the endstage of apoptosis is observed only on day 3 (Figure 2B).
- In order to determine whether this new phenomenon of hDNA-induced massive apoptosis is dependent on a particular body system, similar experiments were carried out using ascitic cells isolated from animals and cultured *ex vivo*. As it turned out, very similar pattern of induced apoptosis in ascitic cells is observed. Cell counts went down when treated with DNA, whereas when ascites were left untreated, cells counts increased (data not shown). Furthermore, *ex vivo* experiments fully recapitulated the *in vivo* effect of conversion of apoptotic DNA (at 24 h) into DNA degradation pattern consistent with secondary necrosis at 72 h (Figure 3С2, 4).

A second series of experiments, when hDNA-only injections were done within 12 hours, allowed us to disentangle the complex picture of events and to uncover a previously unreported phenomenon:

- When using mice with 9-day ascites, they started dying during the course of hDNA-only injections (Figure 1A). This was accompanied with induced apoptosis of ascitic cells. Apoptosis with detectable nucleosomal DNA fragmentation is observed 24 hours after hDNA injection (Figure 3C1). Increase in LDH levels is delayed and is first detectable 48 hours post first hDNA injection (Figure 1B). Notably, at this period of time we find no evidence of DNA cleavage into nucleosomal fragments, as it follows from the agarose gel analysis of DNA isolated from ascites (Figure 3С3). Clearly, this is consistent with complete disintegration of chromatin, which is typical for necrotic phase of apoptosis.

### Induction of apoptosis in MCF-7 cells by human dsDNA preparation

**Figure 4:**
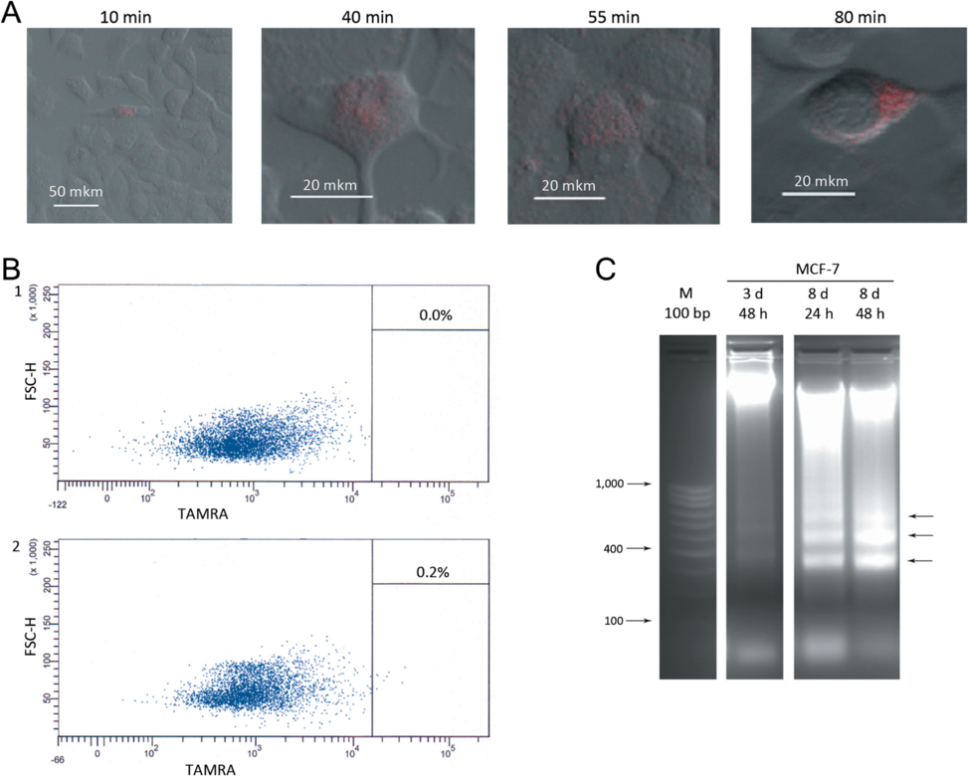
**Analysis of hDNA internalization and apoptosis induction in MCF-7 cell line. А)** Confocal imaging of TAMRA-labeled Alu DNA internalization by MCF-7 cells after 10, 40, 55 and 80 minutes of co-incubation. **В)** Internalization of TAMRA-labeled Alu DNA by MCF-7 cells (flow cytometry analysis): 1 – control cells without DNA; 2 – cells incubated with TAMRA-labeled DNA for 1 hour. **С)** Nucleosomal ladder formed by DNA isolated from MCF-7 cells which were cultured with exogenous hDNA and induced by TNFα to undergo apoptosis. 3 d, 8 d denote 3 and 8 days of co-incubation with DNA, accordingly; 24 h and 48 h indicate the duration of TNFα-induced apoptosis. Arrows point to the typical DNA bands found upon apoptotic DNA defragmentation.

The phenomenon of massive cell apoptosis was originally described by our group in experiments on mouse bone marrow cells: up to 90% of such cells became apoptotic depending on the DNA substrate used [28,29]. These and subsequent experiments established that 1% and 1-7% of nucleated bone marrow cells and Krebs-2 ascites were hDNA-internalizing [30]. This striking difference between low percentage of hDNA-internalizing cells and high percentage of cells undergoing apoptosis may indicate that apoptosis is not linked directly to hDNA internalization. Therefore, some alternative mechanism of apoptosis induction must be in place. We used a third independent cellular model, MCF-7 cell line, to further illustrate the phenomenon of massive induction of apoptosis by hDNA fragments. First, we calculated the percentage of MCF-7 cells capable of internalizing extracellular hDNA fragments. Using fluorescence microscopy and flow cytometry, we established that ~0.2% MCF-7 cells can internalize TAMRA-labeled *Alu* fragment upon co-incubated for 1 hour (Figure 4А,В). To assess the magnitude of apoptosis in MCF-7 cells, these cells were incubated for different periods of time with exogenous DNA and apoptosis was induced by addition of TNF-α. Total DNA was fractionated by gel electrophoresis. Then the DNA was extracted from the gel and quantified. Given that cellular DNA content is known, this could be translated into the percentage of apoptotic cells (see Materials and Methods). The results obtained suggest that the percentage of MCF-7 undergoing apoptosis is at least 14.8% and 44.5%, after 3 days of incubation followed by 48 hours induction and and upon 8 days + 48 h induction, respectively (Figure 4С).

These data prompted us to explore whether dsDNA-induced induction of apoptosis is also characteristic for other cell types, − either produced *ex vivo* or passaged as cell lines. Three distinct cell types were thus analyzed, namely mitomycin-treated mouse fibroblasts (feeder cells), human embryonic kidney 293FT cell line and mouse bone marrow cells produced *ex vivo*. We observed that dsDNA preparation failed to induce apoptosis under these specific conditions (dsDNA/cell ratio was approximately the same as in experiments *in vivo*, 75–150 mg DNA/10^6^ cells) in feeder cells and HEK 293FT cells. In contrast, mouse bone marrow cells become apoptotic, yet this effect is less prominent, as compared to the situation when mice are injected with dsDNA (data not shown).

## Discussion

### Causes of death of experimental animals

Our study firmly establishes that death of animals was caused by a complex contribution of several factors: presence of well-developed ascites, injection of hDNA, CP or combination thereof.

We found that large, 10–15 cm^3^, ascites grown in mice for 9–15 days compromise the integrity of the gut cell wall so that intestinal bacteria invade the peritoneal space. This is accompanied with inflammatory reaction, as was shown for late ascites by Parsons et al. [56]. Nevertheless, the above-mentioned ascites-induced bacterial contamination is relatively subacute, it is spread across the treatments and does not kill lab animals quickly. In contrast, treatment with hDNA or CP + hDNA greatly stimulates and accelerates the destruction of intestinal cell wall. Peritoneal and tumor necrotic foci are formed. Under this scenario, infection of peritoneal space occurs synchronously and massively in different animals, which typically takes 2 days following CP and/or hDNA treatments.

This process is overlayed with the following events that greatly stimulate decomposition of the tumor and adjacent tissues and organs. Human DNA, CP or CP + hDNA treatments induce massive apoptosis of ascitic cells, with rapid progression into secondary necrosis. The onset of apoptosis induced by hDNA is distinct from that of CP-induced apoptosis. The final stage of hDNA-induced apoptosis (chromatin break-down into nucleosomal fragments) is clearly detectable by the end of day one of treatments. CP-induced apoptosis, in contrast, is apparent on day 3 after CP injection. No apoptosis is observed at this time in ascitic fluid upon hDNA-only injection. Significant increase in LDH levels and complete degradation of cellular DNA are typical for this stage, which is consistent with necrotic cellular lysis. Therefore, two distinct mechanisms for apoptotic cell death of ascites are at place, differing in the timing of the final stage of apoptosis.

CP-induced apoptosis is well characterized experimentally, and is associated with aberrant mitosis caused by error-prone repair of double-stranded breaks. It appears as a nucleosomal DNA ladder on days 3–4 after CP injection [57,58].

Molecular underpinnings of hDNA-induced apoptosis still await detailed experimental analysis. Yet, we speculate that the nature of these effects may be similar to the bystander effect. Bystander effect is known to result from oxidative stress, activation of caspases and DNA-sensing receptors [19,59,60]. Extracellular dsDNA has been demonstrated to relay the signal to bystander cells in the following sequence of events. Free radicals induced by gamma-radiation result in primary oxidative stress which leads to apoptosis of irradiated cells. DsDNA fragments liberated from such apoptotic cells interact with DNA-binding receptors of bystander cells. This in turn activates signaling pathways boosting production of reactive oxygen and nitrogen species (in the context of human lymphocytes). This induces secondary oxidative stress leading to apoptosis of bystander cells, which takes 24 hours [19].

Whatever the exact mechanism, the magnitude of this effect prevents the specialized cells of immune system from clearing the peritoneal space from excess of apoptotic bodies. By day 2 following DNA treatment, and by day 4 post CP treatment, secondary necrosis of ascitic cells is well discernible [61-63], which leads to rapid progression into systemic inflammatory reaction [22,62,64-66]. This generalized necrosis may be further enhanced by lymphocyte necrosis, which is caused by the release of nucleosomes from apoptotic bodies [67].

Thus, the death of mice is caused by two major destructive processes. In the presence of a well-developed ascites and upon the combined CP and hDNA treatment, the barrier function of intestinal cell wall is compromised catastrophically quickly, which progresses into bacterial contamination of ascitic fluid and general body sepsis. This effect is accompanied with massive apoptosis and secondary necrosis of ascitic cells, which accelerates and boosts necrosis in peritoneum and gut wall, thereby promoting infection. Massive necrosis culminates in a full-blown inflammatory reaction. The range of pathological changes in the organism of experimental animals shares many common features with both systemic inflammation response syndrome and sepsis known from clinical practice [22,65,66]. In this respect, this system may become a useful model to explore treatment modalities for sepsis, which is frequently a lethal condition.

### Apoptosis of MCF-7 cells and general conclusion on the phenomenon of induced apoptosis

Using a third distinct model, MCF-7 cells, our experiments demonstrated induction of massive apoptosis by fragmented hDNA. Depending on the duration of specific treatments, up to 44% of MCF-7 cells may undergo apoptosis. In the three cell types tested (mouse bone marrow cells, Krebs-2 ascites and MCF-7 cell line), 0.2-7% of cells were shown to internalize hDNA. Yet, the percentage of cells becoming apoptotic in these cell populations ranged between 40 and 90%. Two possibilities may account for this effect. First, the hDNA-internalizing cells may secrete specific factor(s) that launch apoptotic signaling in the neighboring cells. Alternatively, and somewhat more likely, the DNA fragments could be recognized by specific cytoplasmic sensors/receptors harboring “death domains” that in turn induce apoptosis via mitochondrial pathway.

In our additional experiments on different cell lines, nucleosomal fragmentation of chromatin was detectable in only a subset of cell lines tested. Specifically, no apoptosis could be detected in HEK 293 FT cell line. In contrast, mouse bone marrow cells underwent apoptosis both *in vivo* [28] and upon culturing *ex vivo*. No microscopically detectable apoptosis was observed in mouse and human DCs produced *ex vivo* [51-53,68,69].

Taken together, these data suggest that dsDNA-induced cell apoptosis is not a universal feature of all cells, and its induction depends on the biology of specific cell type tested.

## Conclusions

The present study demonstrates that hDNA, CP or CP + hDNA treatments accelerate the destruction of intestinal cell wall in animals with late ascites. This is due to massive apoptosis of ascites cells followed by secondary necrosis by day 2 for hDNA and by day 4 for CP treatment, which results in systemic inflammatory reaction and body sepsis in experimental mice. MCF-7 cells treated with hDNA preparation likewise demonstrate extensive apoptosis. There are two main plausible mechanisms underlying the effect obtained, namely the “bystander effect” and specific recognition of DNA fragments by cytoplasmic sensors, but the exact nature of the processes involved still remains to be determined.

## Methods

### Lab animals

We used 2-8-month old CBA/Lac, CC57BR and C57Bl mice strain bred in the Institute of Cytology and Genetics, SB RAS. Animals were grown in groups of 5–10 mice per cage with free access to food and water. All experiments were performed in accordance with protocols approved by the Animal Care and Use Committee of the Institute of Cytology and Genetics.

### Tumor model

Murine Krebs-2 tumor model was used. To obtain ascites, Krebs-2 cells were diluted 1:10 in 200 μl of normal saline and inoculated i.p. (~2 × 10^6^ cells). To obtain solid tumors, Krebs-2 cells were engrafted intramuscularly into the right hind leg in 100 μl of RPMI-1640 or PBS. In course of experiments, as soon as tumors became palpable (about 7 days following the inoculation), they were measured every 1–2 days with calipers, and tumor volume was calculated as follows: volume = length × width × height.

### Injection of CP and exogenous DNA preparations

I.p. or intravenously injections of a cytostatic drug CP were administered to mice at a dose of 100 or 200 mg/kg body weight. Next, depending on the specific experimental design, the preparations of fragmented human DNA (hDNA) or nitrogen mustard-treated DNA (ICL-hDNA) were injected i.p. or directly into the solid tumor at 0.5 mg/injection. The following injection schedules were used: hourly 1–12 hrs or 18–30 hrs after CP injection (i.e. a total of 6 mg hDNA prep/mouse). As a control, an equal volume of normal saline (200 μl/injection) was used. hDNA injection into solid tumor was done into multiple sites of a tumor during each injection.

### Analysis of apoptotic degradation of Krebs-2 ascites cells

Apoptotic degradation of cells was analyzed via agarose gel electrophoretic separation of DNA isolated from cells or ascitic fluid.

#### Isolation of DNA from ascitic fluid

Post-treatment ascites were collected and centrifuged at 400 g (Eppendorf 5810 R), at 4°С for 5 minutes. EDTA (Sigma) and proteinase K (Medigen) were added to 500 mkl of supernatant to a final concentration of 0.5% and 100 mkg/ml, respectively, and samples were incubated for 1 hour at 58°С. Next, an equal volume of phenol-chloroform (1:1) was added, gently mixed and centrifuged at room temperature at 3200 g (Eppendorf 5810 R) for 10 minutes. Upper aqueous phase was transferred into new tubes and re-precipitated by adding 0.1 volume of 3 M NaAc and 0.6 volumes of isopropanol. The cloudy pellet was left to form on ice for 10 minutes and spun down at 4°С, 3200 g, 15 minutes. Supernatant was decanted, the pellet was washed with 70% ethanol and dissolved in 50–100 mkl autoclaved water.

#### Isolation of DNA from ascites

The collected ascites were washed in 1 ml of PBS (Medigen), and centrifuged at 400 g (Eppendorf 5810 R), 4°С for 5 minutes. Cell pellet thus obtained was resupended in 2 ml PBS, EDTA was added to 20 mM, followed by SDS to 1% and proteinase K to 200 mkg/ml. The suspension was incubated for 2 hours at 58°С and nucleic acids were phenol-chloropform (1:1) extracted as above. After re-precipitation and washes, the pellet was dissolved in 150 mkl water.

### Quantification of LDH levels in ascitic fluid

Ascites were collected on day 3 following the treatments and cells were pelleted by centrifugation at 400 g at 4°С for 5 minutes. Supernatants (50 μl) were transferred into clot activator vials and sent out for analysis at Siblabservis, Ltd (Novosibirsk, Russia).

### TAMRA labeling of human Alu repeat DNA

DNA was labeled using PCR. PCR template was human *Alu* repeat material cloned in pUC19, this repeat encompassed the start and the end of tandemly repeated *AluJ* and *AluY* (NCBI: AC002400.1, 53494–53767). Standard M13 primers were used for amplification. PCR purification was done by phenol-chloroform extraction followed by ethanol precipitation using ammonium acetate as a salt. DNA concentration and incorporation of dUTP-TAMRA were measured using Nanodrop (Eppendorf) and calculated by comparing the signal before and after PCR re-precipitation.

### DNA preparation

Human DNA preparations were isolated from placentas of healthy women using a phenol-free method. DNA was fragmented in an ultrasonic disintegrator at a frequency of 22 kHz to obtain a mixture of DNA fragments with a size of 200 to 6000 bp. DNA preparations were dissolved in saline and stored at −20°C. This is a pharmacopieal drug (Registration certificate Medical Drugs of Russia No. 004429/08 of 09.06.2008) and is manufactured under a trademark of Panagen, Ltd.

### Preparation of nitrogen-mustard cross-linked DNA preparation

Cross-linked human DNA (ICL-hDNA) was obtained by incubating 500 mg hDNA in a reaction mixture of 200 mM sodium-phosphate buffer, pH 7.4, 400 mkM mechlorethamine (Merck) in a volume of 1 L adjusted with water for 24 hours at room temperature [70,71].

Next, the DNA was re-precipitated by addition of NaAc pH 5.3 to final concentration of 0.3 M and 0.6 V isopropanol and dissolved in 1 L of water. Cetavlon (Serva) was added to 2%, and the sample was spun down at 15000 g (Beckman J2-21) for 40 minutes at room temperature. Supernatants were decanted and centrifuge tubes were dried up-side-down for 5 minutes on filter paper. The pellets were mixed with 200 ml 5% cetavlon and centrifuged at 25000 g, for 30 minutes at room temperature. Supernatants were removed by decanting and tubes were air-dried for 5 minutes on a filter paper. In order to assure that no phosphate buffer remains, cetavlon treatment was repeated for one more time.

Air-dried pellet was completely dissolved in 150 ml ethanol by incubating at 65°С for 40 minutes. Then, 3 ml 5 M NaCl was added and samples in centrifuge flasks were put at −20°C overnight. The samples were centrifuged at 25000 g, 20 minutes at +4°С, and the pellet was carefully dried by placing the tube on a filter paper. The pellet was mixed again with 200 ml of ice-cold ethanol, centrifuged and air-dried as above, followed by drying for 10 minutes at 37°С. DNA pellet was dissolved in 45 ml of bi-distilled water. The yield of nitrogen mustard-treated human DNA was 486 mg, with size range of DNA fragments remaining unchanged.

### Histopathology analysis of mouse tissues and organs

Pieces of tissues, organs, tumor foci and peritoneal wall from mice engrafted with ascites were fixed in 4% formaldehyde, dehydrated in a graded series of alcohols, cleared in xylol and embedded in paraffin. 5 μm thick paraffin sections were stained with hematoxylin and eosin. AxioImager ZI microscope (Zeiss) was used for imaging.

### Sterility culture

Ascitic material collected from the treated animals was centrifuged at 400 g (Eppendorf 5810 R), 4°С for 5 minutes. Supernatant was spread on agar plates. Each plate contained a mixture of ascitic fluid from 5 animals (250 mkl total).

### MCF-7 cell culture

Human breast adenocarcinoma (MCF-7) cells were obtained from the Institute of Cell Cultures, SRC VB Vector (Koltsovo, Novosibirsk oblast, Russia) and tested for the absence of mycoplasma contamination. Authenticity of the culture was confirmed by DNA fingerprinting and cytological examination. The cells were cultivated in the RPMI-1640 medium supplemented with 10 mM L-glutamine and 50 μg/ml streptomycin (Sigma) at 37°C in the presence of 5% fetal bovine serum (Biolot) in an atmosphere of 5% CO_2_ at 70-100% confluency.

### Fluorescence microscopy and flow cytometry analysis of internalization of TAMRA-labeled hDNA by MCF-7 cells

MCF-7 cell culture was grown to 80-90% confluence. 0.02 mkg TAMRA-labeled human *Alu* DNA was added to 1.8 × 10^6^ MCF-7 cells (ø40 mm Petri dish) and immediately placed under laser scanning microscope LSM 510 META (Zeiss).

5.4 × 10^6^ MCF-7 cells were incubated with 0.06 mkg TAMRA-labeled human Alu DNA for 1 hour at 37°C. Then the cells were trypsinized and washed once with PBS. Internalization of labeled DNA was assayed using flow cytometer BD FACS Aria (BD, USA), with cells not incubated with DNA serving as a negative control.

### Isolation and analysis of apoptotic DNA

To analyze the DNA fragmentation during apoptosis, MCF-7 cells were incubated for 24 h or 48 h at 37°C in the presence of 0.1 μg/ml tumor necrosis factor-alpha (TNF-α) and 10 μg/ml cycloheximide in RPMI-1640 supplemented with 10 mM L-glutamine, 50 μg/ml streptomycin, and 5% FBS. For DNA isolation, the cells resuspended in water were supplemented with SDS to 1%, EDTA to 20 mM, and proteinase K to 200 μg/ml and incubated at 60°C for 1 h. DNA was precipitated with isopropanol, dissolved in water, again adjusted with EDTA to 20 mM and proteinase K to 200 μg/ml and incubated at 60°C for 3 h. The mixture was supplemented with 500 μg/ml ribonuclease and kept for 60 min at 37°C. The specimens were analyzed in 2% agarose gel by electrophoresis.

To assess the progression of apoptosis, four most prominent DNA bands of apoptotic ladder (180, 360, 540 and 720 bp) were excised and gel-purified. DNA concentration was measured using Nanodrop (Eppendorf). These DNA fragments represent the final products of DNAse-mediated degradation of genomic DNA. The weight of these DNA fragments equal to the weight of a single cell genome can be considered as a weight of DNA of a single cell completely hydrolyzed down to these four monomers. Hence, the weight of these four DNA bands divided by the 2n genome weight (~6 pg) translates into the minimal number of cells that achieved complete apoptotic degradation, i.e. it corresponds to the minimum % apoptosis (Table 1).

**Тable 1:** **Analysis of MCF-7 cells becoming apoptotic following culturing with fragmented exogenous human DNA and induced to apoptosis with TNFa**

**DNA fragment**	**Concentration, ng/mkl**	**Weight, mkg**	**Length, bp**	Cell number, *10 ^**6**^	**Percentage of apoptotic cells**
1,0	15.3	0.77			
1,1	18.3	1.83			
1,2	10.6	0.53	720	0.074	9.3
1,3	13.6	0.68	540	0.095	11.9
1,4	19.1	0.96	360	0.135	16.9
1,5	7.6	0.38	180	0.053	6.6
Lane 1	5.15			0.8	44.7
2,0	110.9	5.55			
2,1	14.7	1.47			
2,2	4.2	0.21	720	0.029	2.2
2,3	3.4	0.17	540	0.024	1.8
2,4	8.1	0.41	360	0.057	4.4
2,5	11.7	0.59	180	0.083	6.4
Lane 2		8.4		1.3	14.8

1,0, 2,0 – genomic DNA, 1,2-1,5, 2,2-2,5 – four most fast-migrating nucleosomal fragments, 1,1, 2,1 – larger fragments of apoptotic DNA.

### Statistical analysis

Students’ t-tests were used to determine the significance of differences in tumor growth, and average survival between the mouse groups and the controls. All results are expressed as mean ± SEM.

Absence of SEM “whiskers” on the plots denotes that only one or two mice survived to this experimental time point, thereby precluding statistical analysis. Nevertheless, these datapoints are presented, as they demonstrate the maximum toxicity effect of the treatments performed.

Statistical significance of differences in LDH levels was assessed using non-parametric statistics and Mann–Whitney U-test.

## Additional file

Additional file 1:
**Pathomorphology analysis of mouse organs and tissues.**

## Abbreviations

CP: Cyclophosphamide
dsDNA: Double-stranded DNA
hDNA: Human DNA
ICL: Interstrand cross-link
