# Supplementary material for: Combination of cyclophosphamide and double-stranded DNA demonstrates synergistic toxicity against established xenografts
Source: Cancer Cell Int. 2015 Mar 19;15:32. doi: 10.1186/s12935-015-0180-6 (PMC4369063; doi:10.1186/s12935-015-0180-6)
Supplement: Additional file 1: — Pathomorphology analysis of mouse organs and tissues. [file 12935_2015_180_MOESM1_ESM.pdf]

Additional file 1 – Pathomorphology analysis of mouse organs and tissues

CP+ICL-hDNA (1-12) group

Pathological changes in liver were well-pronounced and occasionally appeared as necrobiosis. Such regions displayed loss of cellular borders between hepatocytes and complete disruption of their cytoplasmic organization. They appeared as homogeneous eosinophilic staining on the slides **(Fig. 1)**.

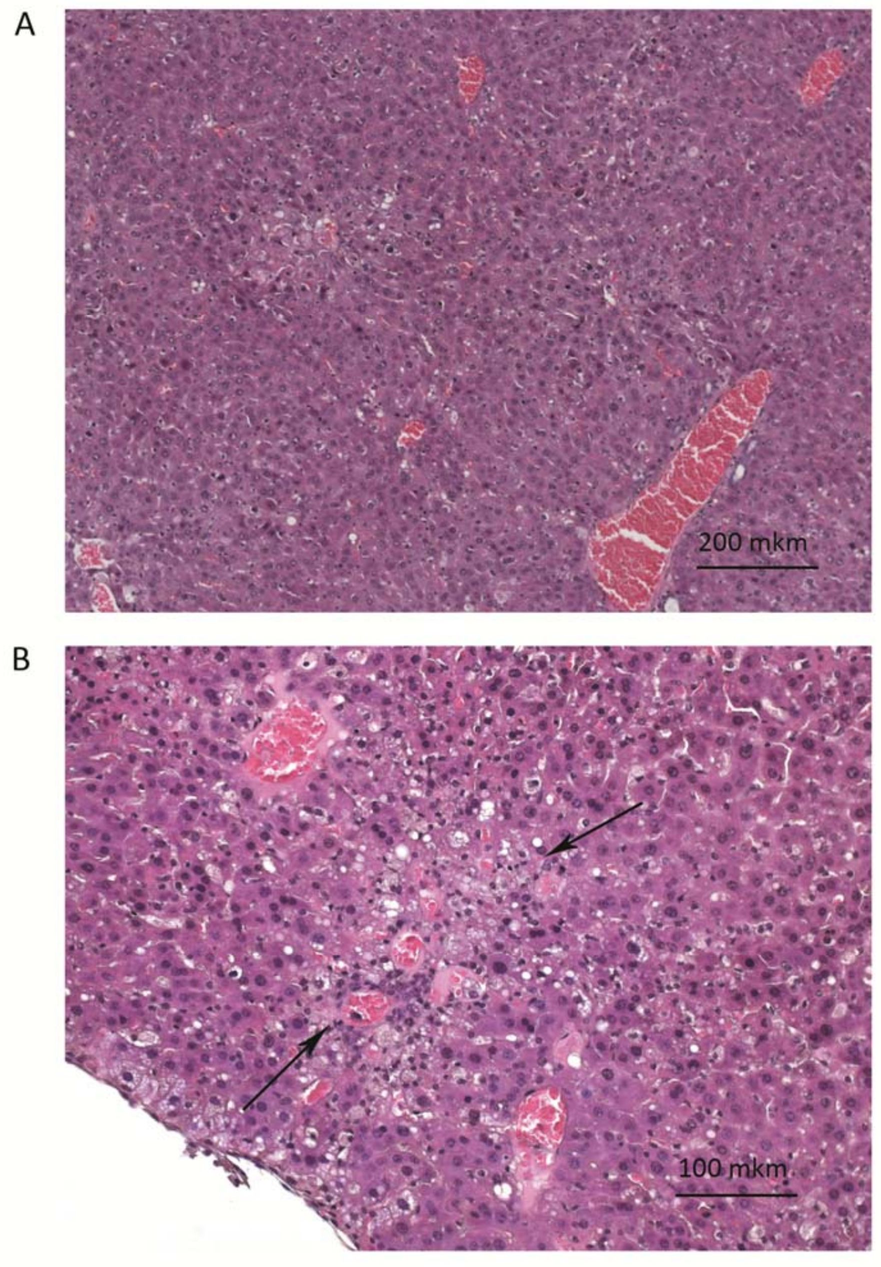

Figure 1. Liver section of a mouse from CP+ICL-hDNA (1-12) group. A) High magnification view. **B)** Site showing necrobiotic changes in hepatocytes (arrows).

In addition, one animal from this group displayed inflammatory changes in lungs and spleen.

Examination of *lung* sections showed focal pneumonia **(Fig. 2)**. Notably, in two out of three animals in the group the lesions were quite large.

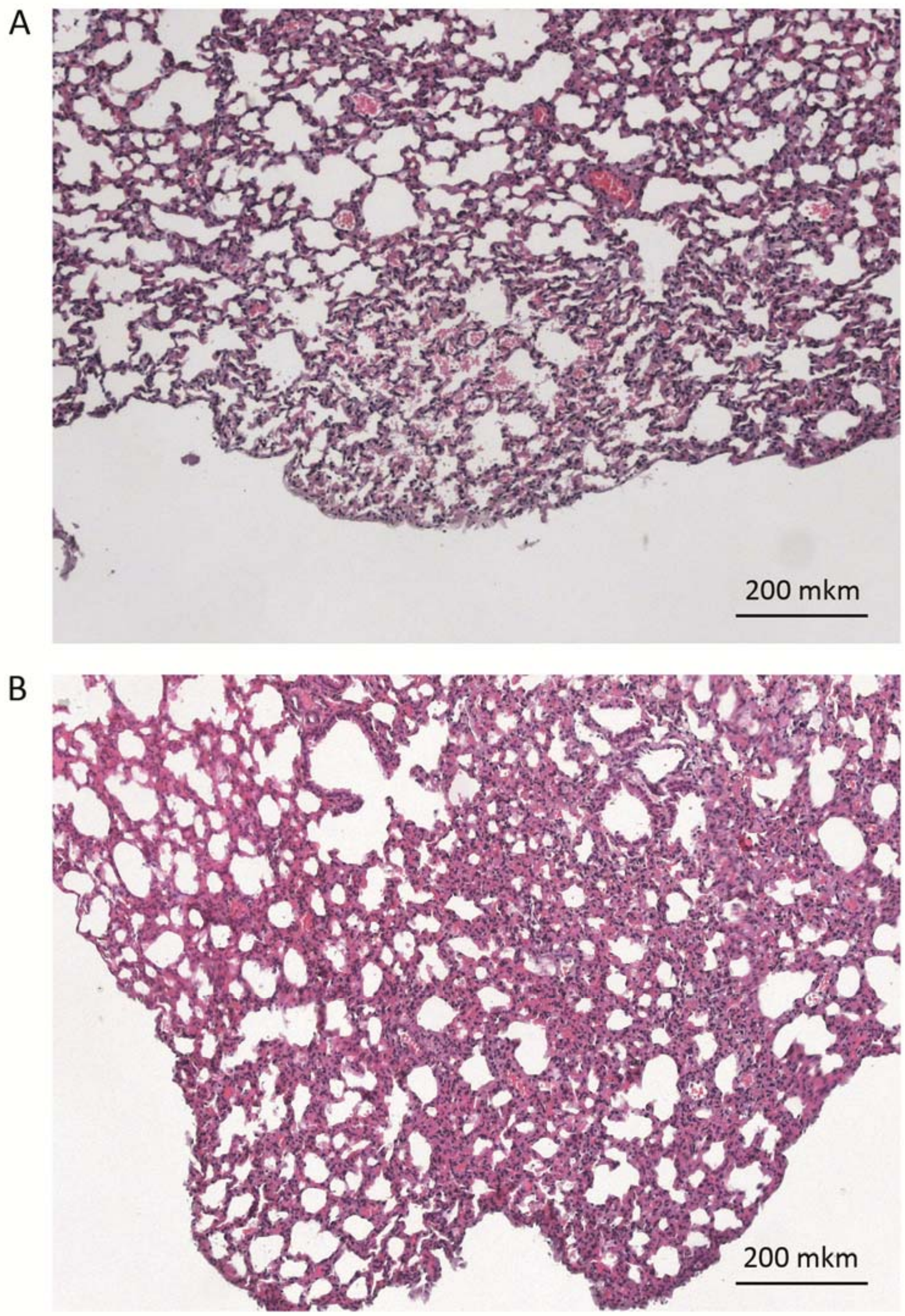

Figure 2. Lungs section of a mouse from CP+ICL-hDNA (1-12) group. **A)** Close-up view. **B)** Extensive diffuse inflammatory cell infiltrate in parenchyma.

Spleen of two out of three animals showed evidence of splenic pulp necrosis **(Fig. 3A)**. Also, tumor cells were present along the capsular border, but displayed no evidence of spreading into the organ **(Fig. 3B)**.

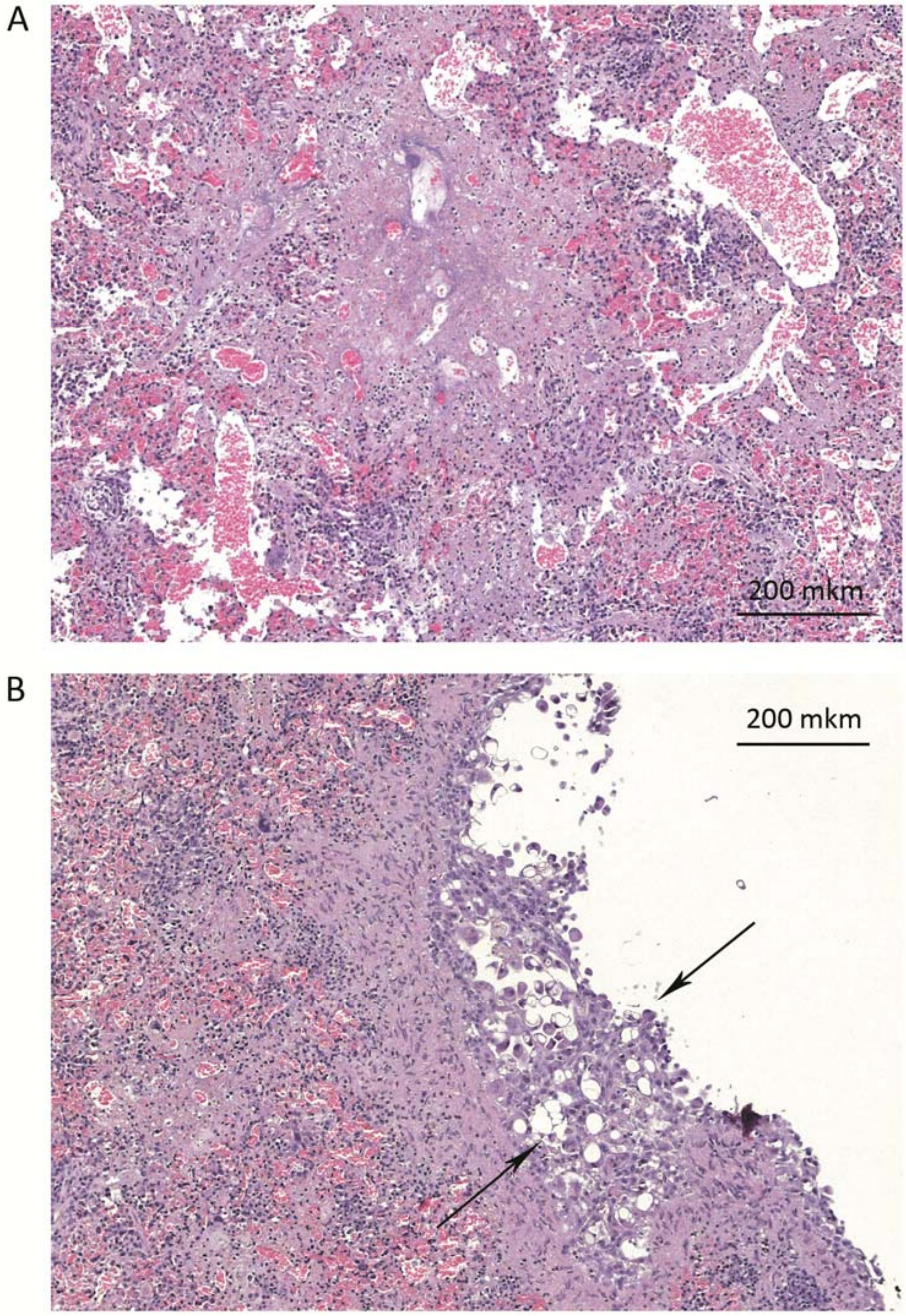

Figure 3. Spleen section of a mouse from CP+ICL-hDNA (1-12) group. **A)** Splenic necrosis. **B)** Accumulation of tumor cells attached to the splenic capsule (arrow).

CP+ICL-hDNA (18-30) group

Two out of seven animals in this group displayed most dramatic changes in the *liver* appearing as necrobiotic foci **(Fig. 4)**.

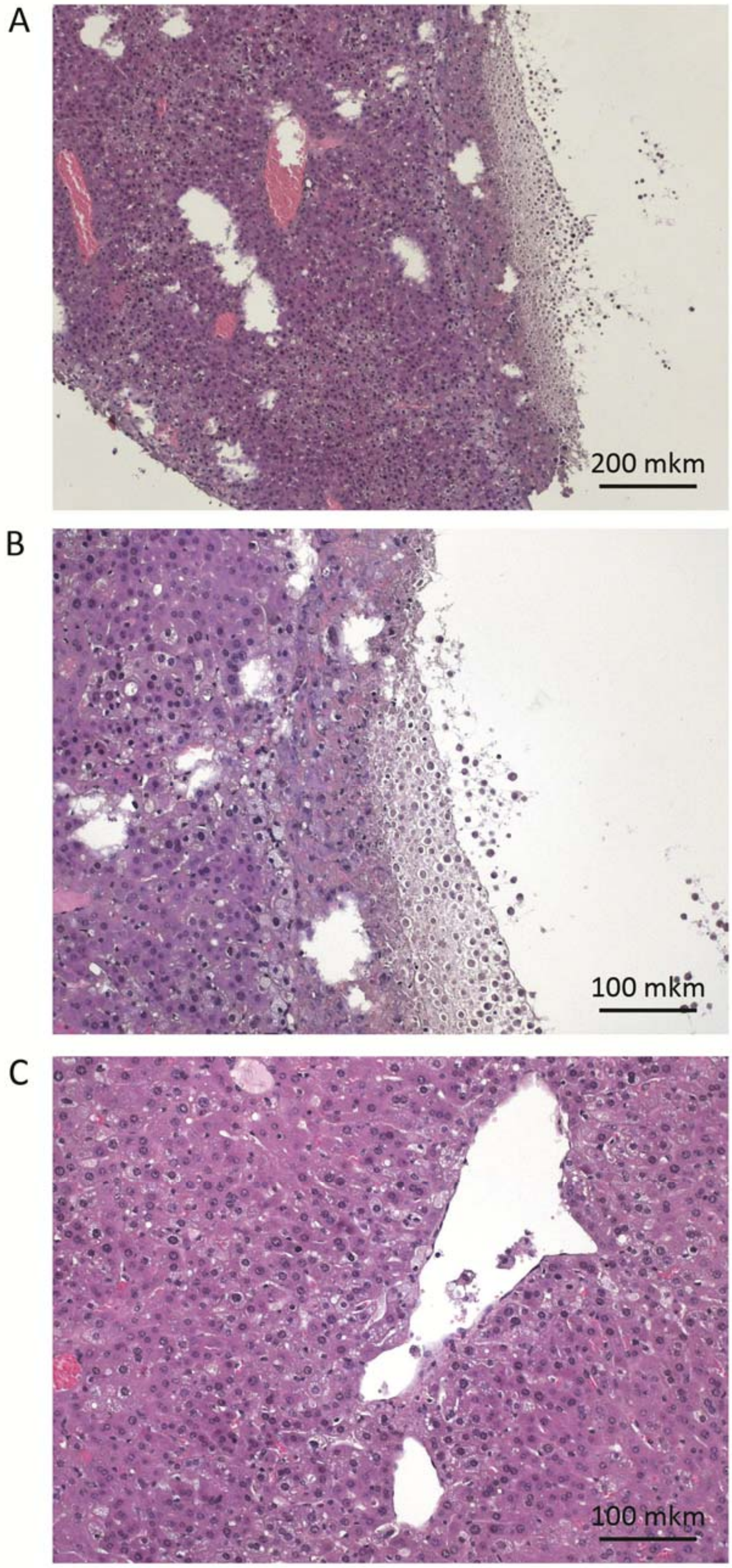

Figure 4. Liver section of a mouse from CP+ICL-hDNA (18-30) group. **A)** Tumor cells lining the outer surface of Glisson's capsule. **B)** Zoom-in of a region showing in **A**. **C)** Diffuse vacuolar dystrophy of hepatocytes.

In one animal, necrobiotic changes were accompanied with inflammatory foci in the lungs **(Fig. 5)**.

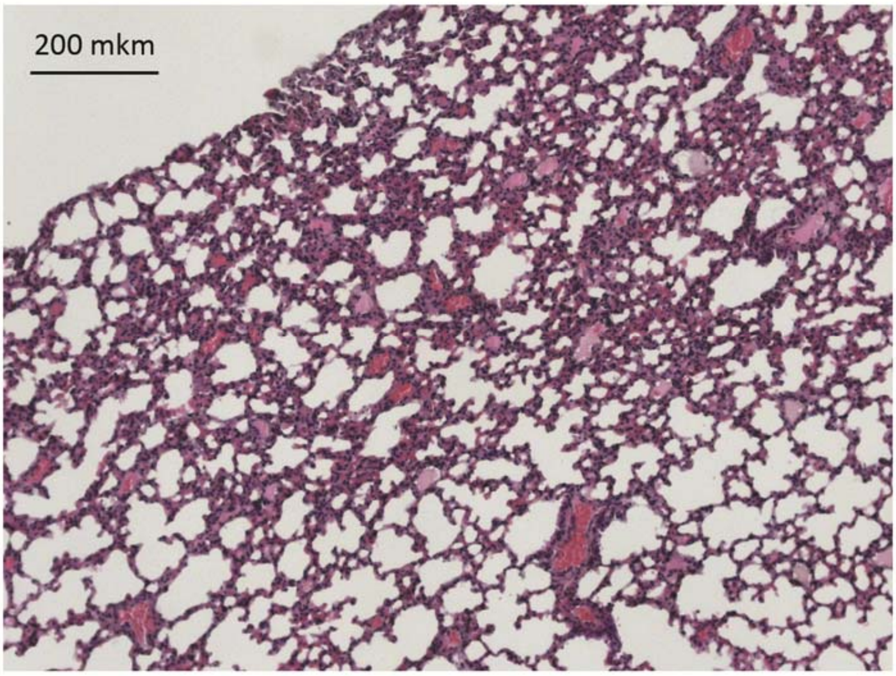

Figure 5. Lungs section of a mouse from CP+ICL-hDNA (18-30) group. Pneumonitis focus.

The rest of the organs displayed pathology changes similar to the ones described above **(Fig. 6, 7, 8, 9)**.

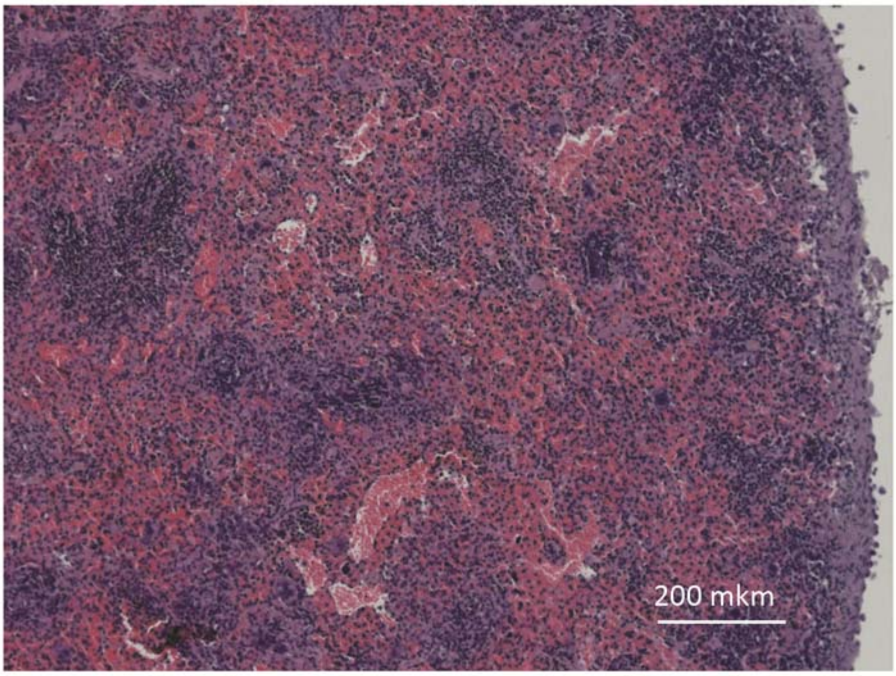

Figure 6. Spleen section of a mouse from CP+ICL-hDNA (18-30) group. Splenic necrosis.

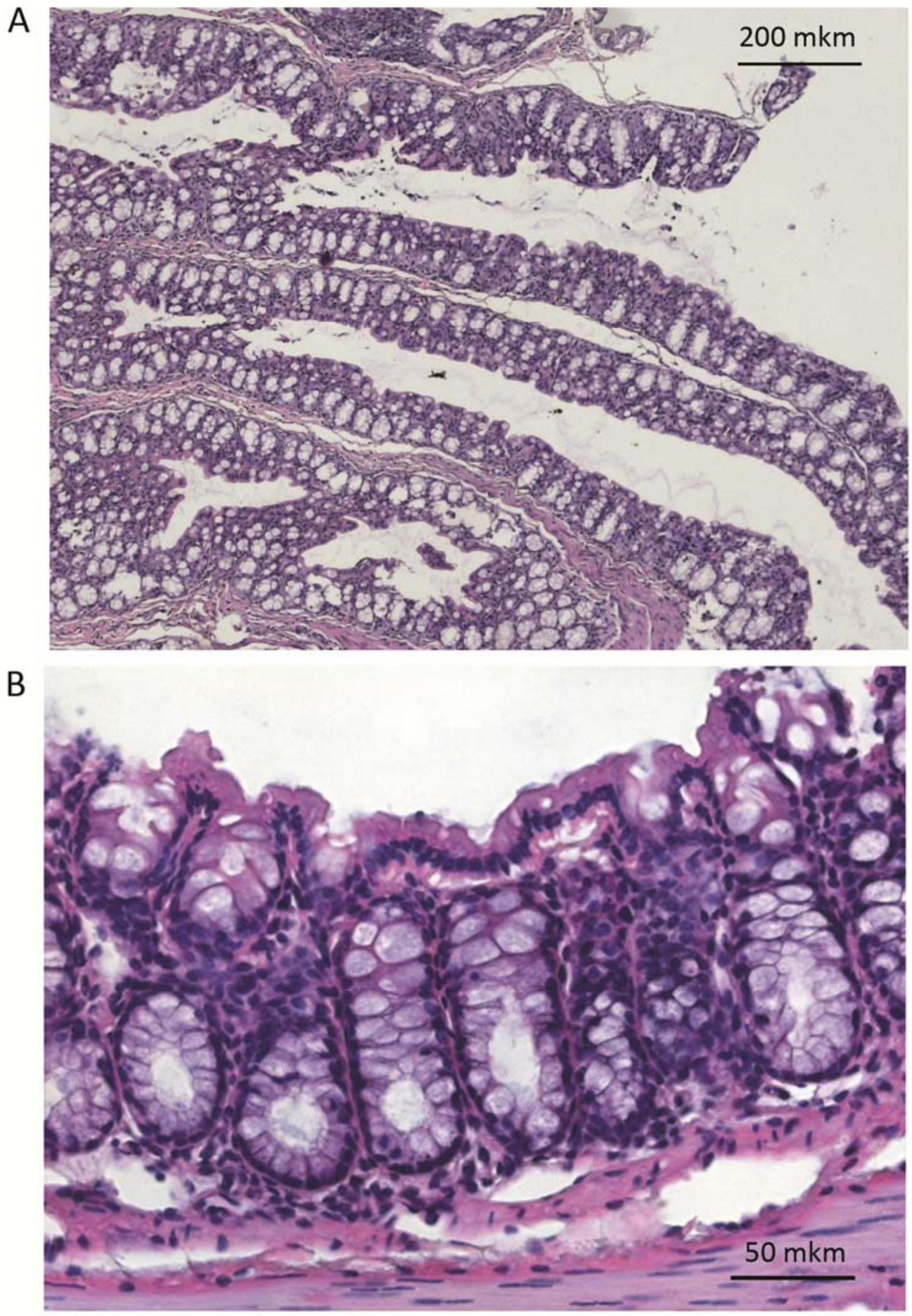

Figure 7. Large intestine section of a mouse from CP+ICL-hDNA (18-30) group. **A)** Large intestine of an experimental animal (no pathological changes whatsoever). **B)** Normal large intestine.

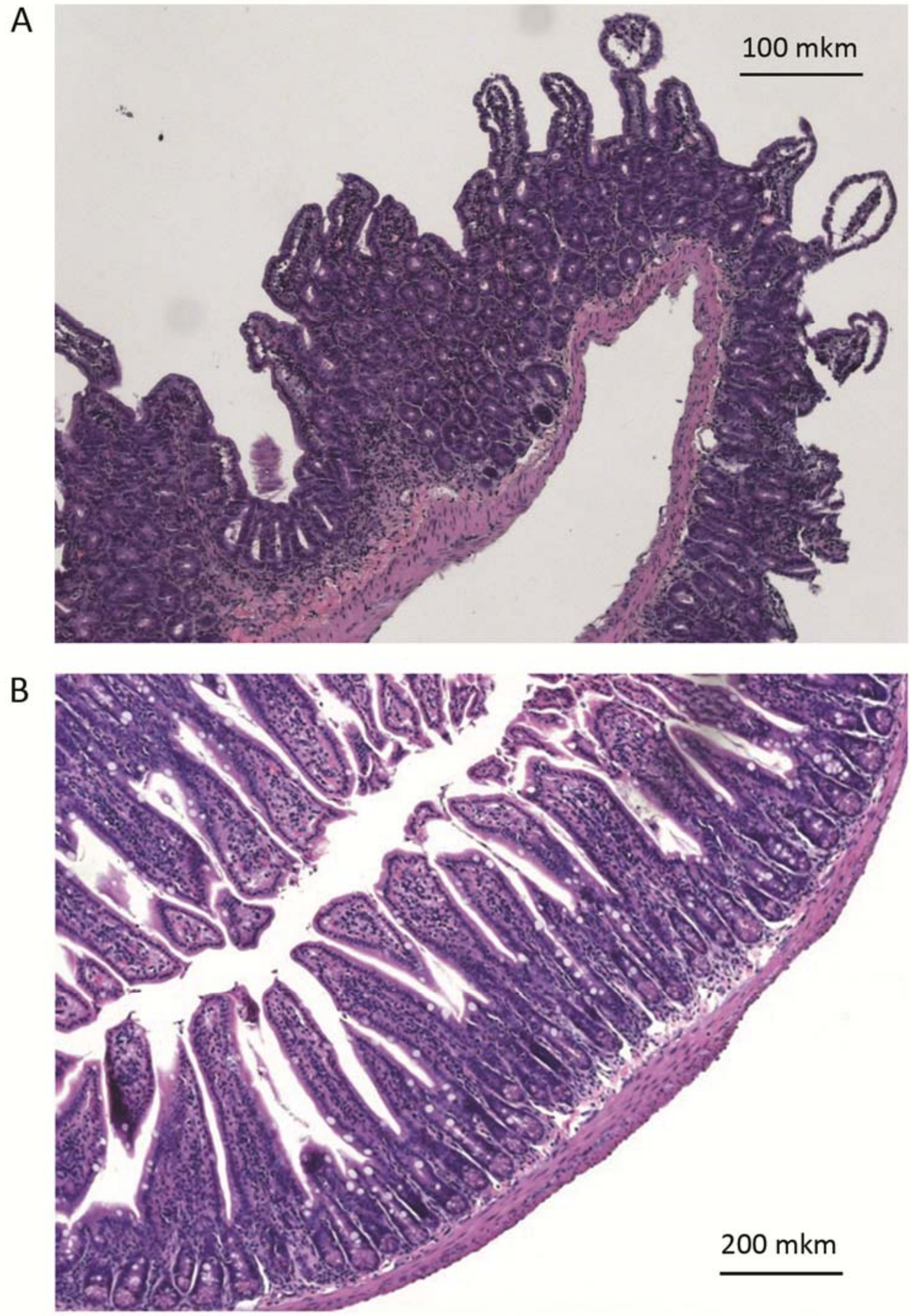

Figure 8. Small intestine section of a mouse from CP+ICL-hDNA (18-30) group. **A)** Intestinal villi appear eroded, epithelial lining is largely gone, pronounced inflammatory cell infiltration combined with connective tissue overgrowth. The changes described correspond to morphological features of chronic enteritis. **B)** Normal small intestine.

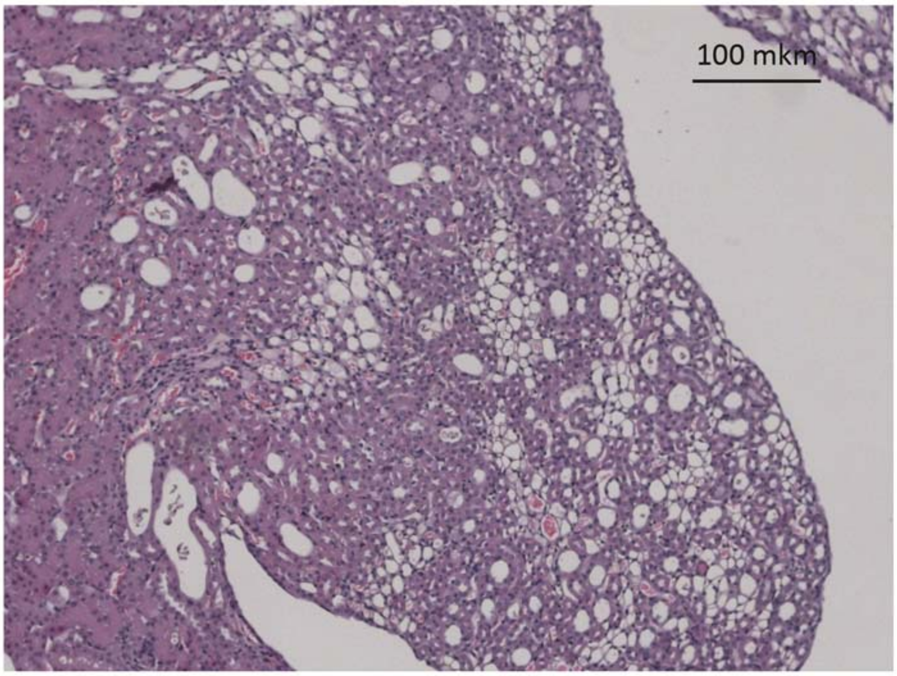

Figure 9. Kidney section of a mouse from CP+ICL-hDNA (18-30) group. No pathological changes.

Several pathological markers are prominent in CP+ICL-hDNA (1-12) and CP+ICL-hDNA (18-30) groups of mice: these include lesions in liver and spleen, as well as involvement of lungs in several animals.

The severity of liver and spleen pathology is the highest in the CP+ICL-hDNA (1-12) group, where it ranges up to necrosis.
